# What Drives Food Insecurity in Western Australia? How the Perceptions of People at Risk Differ to Those of Stakeholders

**DOI:** 10.3390/nu10081059

**Published:** 2018-08-09

**Authors:** Lucy M. Butcher, Maria M. Ryan, Therese A. O’Sullivan, Johnny Lo, Amanda Devine

**Affiliations:** 1 School of Medical and Health Sciences, Edith Cowan University, Joondalup, WA 6027, Australia; t.osullivan@ecu.edu.au (T.A.O.); a.devine@ecu.edu.au (A.D.); 2 Foodbank Western Australia, Perth Airport, WA 6105, Australia; 3 School of Business and Law, Edith Cowan University, Joondalup, WA 6027, Australia; m.ryan@ecu.edu.au; 4 School of Science, Edith Cowan University, Joondalup, WA 6027, Australia; j.lo@ecu.edu.au

**Keywords:** vulnerable groups, food poverty, food insecurity, food literacy, public health, socioeconomics

## Abstract

Food insecurity is considered a “wicked” problem due to the highly complex and at times undefined casual factors. Although many stakeholders are working to address the problem, a possible divergence exists between their views on food insecurity and those of the people who are actually experiencing the problem. The purpose of this study was to investigate whether there was a difference between the opinions of those “at risk” and stakeholders. A total of seven focus groups (two stakeholder groups *n* = 10, five “at-risk” groups *n* = 34) and three interviews (stakeholders *n* = 3) were conducted to ascertain perceptions. Thematic analysis generated 329 (209 “at-risk” and 120 stakeholder) coded statements related to food insecurity drivers. Respondents were in agreement for the majority of factors, and limited income was considered the primary driver of food insecurity. However, there were notable deviations in the perceived importance of certain drivers, particularly around the price of food and the lack of food literacy. Differences in the perception of causes of food insecurity may in part be attributed to the varied role each group plays in working towards the resolution of the problem, either at the household or system level.

## 1. Introduction

Food security is broadly defined as when all people, at all times, have sufficient food to meet their needs. It is underpinned by four pillars: food availability, access, utilisation and stability [1]. Availability refers to sufficient quantities of appropriate food, access refers to having economic and physical resources to obtain appropriate foods for a nutritious diet, utilisation is knowledge of basic nutrition and cooking skills, and stability refers to continued access that can withstand climatic or economic disasters or seasonal events [1]. Ultimately, the disruption of any of the abovementioned pillars may be the catalyst for food insecurity [2,3]. Often oversimplified as an issue of poverty, the problem remains largely hidden in developed countries such as Australia. The reported Australian prevalence of food insecurity varies considerably, depending on the definition and measurement used, between 4% [4] and 36% [5]. 

Food insecurity is considered a “wicked” problem due to the vast, highly complex and at times undefined casual factors [6]. Part of the challenge of making a tangible difference is the dynamic nature of the issue. Changes to the political, economic and social environments have a flow-on effect on the pillars of food security and thus affect a range of sectors within a population [7]. Although many stakeholders employed in the food relief sector are working to address the problem, a possible divergence exists between their views and those of food-insecure people [8]. Deviations in the perceived underlying casual factors between these two key groups may further complicate the process of resolving the issue of food insecurity. The purpose of this study was to compare and contrast opinions on household and systems level food insecurity drivers by those “at risk” and by stakeholders in Western Australia. To our knowledge, this study was the first to investigate the potential differences of opinions between these two key groups within an Australian context. 

## 2. Materials and Methods 

### 2.1. Sample Groups and Recruitment 

Two groups were purposefully targeted for inclusion in either focus groups or in-depth interviews. The first group included stakeholders, who worked in the area of food insecurity (from government, academic and not for profit sectors) and acted as an expert source of information. The second group consisted of individuals who were determined to be at high risk of food insecurity, termed “at risk” (AR), in order to provide contextual information about the lived experience. Initially, stakeholders were recruited through an email sent to a professional interest group. Snowball sampling and word of mouth were utilised to increase recruitment, as recommended for studies of “hidden” issues [9].

A review of adult (≥18 years old) groups most at risk of food insecurity in Australia has identified tertiary students [10], individuals living in regional and remote areas [11], Aboriginal people [12], refugees [13], Culturally and Linguistically Diverse (CALD) people, low-income earners [14], single parents [15], the homeless [16] and those with chronic medical conditions [17]. The tertiary student groups were recruited via Facebook, posts on university message boards and personal invitations at lectures or conferences. Stakeholders were asked for recommendations or referral to other organisations that could provide access to groups whom they considered to be at a high risk of food insecurity. Individuals who met one or more of these criteria were targeted for inclusion in this study. Simplified definitions of food security and food insecurity were explained to all participants. The research topic was framed, during recruitment, as exploring the reasons why some people can’t access enough or the right types of food in Australia. A $20 food voucher was offered to the AR respondents as compensation for their time and effort. 

### 2.2. Data Collection

Data collection was conducted between December 2015 and November 2016. A semi-structured format, considered appropriate for open, in-depth discussion of sensitive topics with vulnerable groups [18], was utilised. Both respondent groups were offered inclusion via either focus group or interview. An interview guide with prompts was developed for the stakeholder and AR groups. The guide had the same underlying research aims, and questions, but was worded differently to ensure appropriateness for each group. All questions were open-ended and included the following topic areas: food insecurity prevalence, food affordability, barriers and personal experience. The content of the guide was informed by current literature and consultation from stakeholders and co-authors. The interview guide and research questions are available in the Supplementary Materials. 

Individual interviews were also offered to both stakeholders and the AR individuals as an alternative to focus group participation. This interview format was intended to enable flexibility around attendance and to allow discussion of sensitive topics in a more private setting. All focus groups and interviews were conducted in Perth, Western Australia, but included participants who worked or had lived in regional or remote areas of the state. 

### 2.3. Data Analysis 

The Clarke and Braun [19] thematic analysis framework guided the identification of concepts and patterns. Conversations were recorded with permission and transcribed verbatim. Data was de-identified and transcripts were read twice to check for accuracy prior to being imported into NVivo qualitative data analysis software; (QSR International Pty Ltd., Version 11, 2015, Doncaster, VIC, Australia). NVivo was employed for the thematic analysis to increase the efficiency and trustworthiness of results in this qualitative research [20]. In addition to audio recording of the focus groups and interviews, notes were taken during both to assist the generation of themes and key concepts. Triangulation was employed to increase validity the data collection, quality of coding and rigour of the thematic analysis [20]. Investigator triangulation was achieved by co-authors (1) attendance one or more focus groups, review of the transcripts and audio recordings and (2) joint discussion of themes to ensure consistency. The use of this technique ensured that the multiple perspectives provided by the AR group and stakeholders were interpreted correctly and there was a consensus among multiple investigators [21].

The four pillars of food security and the research question formed the basis for the initial framework for coding. Twenty-one traditionally associated factors of food insecurity were identified in the literature and these were listed as potential nodes. New or emerging concepts relevant to the research questions were also coded into nodes. Themes and sub-themes, within these four pillars of food security, were created though the amalgamation and separation of the nodes. Data saturation point was ascertained when no additional themes were identified, and the generation of new nodes had ceased. Data saturation, when used in conjunction with investigator triangulation, is considered an appropriate and sufficient methodology for qualitative research [22]. Matrix coding queries, word clouds, text enquiries and frequencies were used for the data analysis. 

Edith Cowan University’s Human Research Ethics Committee approved the study (Project Number: 11118). 

## 3. Results

Twenty stakeholders were invited to participate via email. Of those, 13 agreed to participate, five did not respond and two declined. Reasons given included a lack of time or an absence of expertise in food security. A total of seven organisations were emailed the study information letter and AR groups were invited to participate. Three organisations agreed to take part: a homeless refuge, a multi-cultural domestic violence group and a food relief agency, from which 34 AR individuals agreed to participate in the study. Of the four organisations that did not take part, three organisations did not feel their groups were suitable and one did not respond. In all, a total of 47 respondents participated in focus groups or interviews. A total of seven focus groups (two stakeholder groups *n* = 10, five AR groups *n* = 34) and three interviews (stakeholders *n* = 3) were conducted. 

Of the three stakeholders who accepted the offer for an interview, two were face-to-face, and one interview was via telephone. All interested AR individuals opted to participate in a focus group. Two tertiary student focus groups were conducted, one with undergraduate students and one with post graduate students. Participant characteristics are outlined in Table 1 and Table 2. 

**Figure 1:**
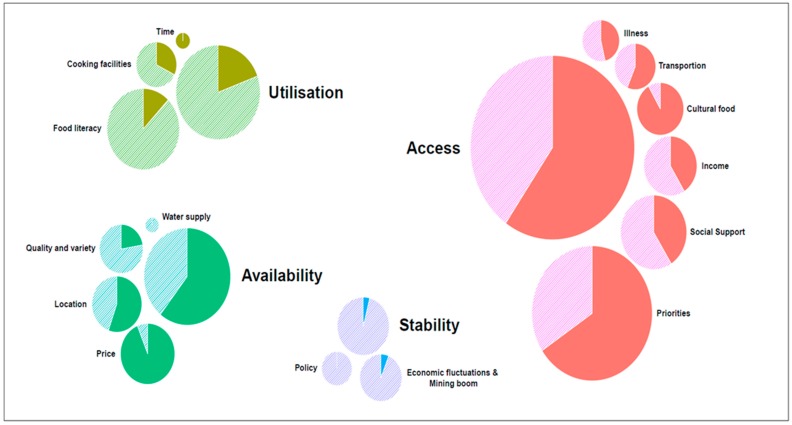
The proportions of the food insecurity drivers cited by stakeholders and at-risk group. The size of the circle indicates the frequency of the driver cited. An even distribution of solid and striped colours indicates that the driver was cited equally by stakeholders and at-risk individuals. A larger proportion of solid colour indicates that the driver was more frequently cited by at-risk individuals than stakeholders, and vice versa.

When asked, approximately two-thirds (68%) of the AR individuals and all stakeholders thought food insecurity was a problem in Australia. Thematic analysis generated 378 individually coded statements. Of these codes, 329 (209 AR and 120 stakeholder) were considered as drivers, either positively or negatively affecting an individual’s healthy food acquisition, and were categorised into the four pillars of food security that formed the primary themes. The remaining 49 codes related to determinants of food security have not been included in this paper. Encompassed within these four primary themes were 15 drivers of food insecurity or sub-themes. Both groups noted similar drivers of food insecurity; however, the most apparent difference was the relative importance attributed to each of these factors. Figure 1 is a graphic comparison of the themes and sub-themes cited by both the stakeholders and AR group.

### 3.1. Food Access Pillar

Access was the most frequently referenced pillar (*n* = 211, 64%); 73% for the AR group (*n* = 152) and 49% for the stakeholder group (*n* = 59). From both groups, six subthemes were identified within the Access pillar: income, priorities, social, cultural foods, transport, and illness and mobility. 

#### 3.1.1. Income

Unexpected bills or costs added an extra burden on already strained finances. AR respondents felt there was no flexibility in the budget for luxuries and that food relief, theft and their social support networks were sometimes used as a means to access additional food. Both respondent groups discussed the concepts of household budget elasticity; competing costs and increasing daily compromises to ensure food supply.

“It relates back to money, but living week to week. If you don’t have savings and you have used all your money for the week. You’ve got $10 left and your food is going to cost $30 dollars. Then there is that gap.”

Stakeholder, Female, 40–49

*“The self-serve juggle. If you put everything through fast enough they don’t realise you’re not paying everything. I mean you can just put through eleven milk cartons when you’re only paying for three. You can do that kind of thing just to save money”*.

At-risk male, 20–29

#### 3.1.2. Priorities 

When income is limited, extensive prioritisation by food-insecure individuals was required to try to ensure perceived basic needs were met. Several compromises were reported by AR respondents and observed by stakeholders: including decreasing the variety of food or buying “cheap” unhealthy options to prevent hunger.

“It’s about how can I make this last and in the home that there are still three meals being placed on the table. The quality of the meal they have also has to be in question as part of that food insecurity. Yes, it lacks any nutritional value, but there are three meals on the table…”

Stakeholder, Female, 50+

All of the AR respondents acknowledged that eating nutritious food and their health was important; however, avoiding hunger was their principal focus. One respondent conceded that they had stolen from supermarkets as means to feel full.

“All your health foods like fruit, veggies and nuts are quite expensive. A piece of bread and Vegemite might suffice on a particular day … You know it’s not great over the long term, but in the short term it’s cheap. It’s not sustaining your health, but it is sustaining your need for food.”

At-risk female, 50+

Housing and essential bills were items prioritised by the AR group; and for several AR individuals, so were vices such as alcohol, drugs or cigarettes.

“Well if I only have so much money, I would rather have cigarettes than food. I’ll be honest with you.”

At-risk female, 50+

#### 3.1.3. Social Network

Both stakeholders and the AR group identified that a person’s social network could have either a positive or negative affect on food security status. Families or group dwellings could share food, transport and resources, ultimately reducing financial burden and potentially increasing the enjoyment associated with food and meal preparation.

“If there were more people in the house the money would share as well. And I feel like if I did cook a big dish ... Then I would have to have left overs for the rest of the week and I would just get sick of it and I wouldn’t want that dish anymore.”

At-risk male, 20–29

Participants reported that living in shared housing or in emergency accommodation meant food could be stolen, or they felt obligated to feed other people who were not able or willing to share the expense. For Aboriginal communities, the transient nature of their society and strong extended family ties made meal planning and budgeting challenging, as it was difficult to predict the size of a household week to week.

*“We have Aboriginal clients here … There are lots of funerals in their families, but every time you have a funeral you have to feed everyone. Often you will get food insecurity if there have been a number of deaths in the family. Families stay after the funerals, so you have to feed all these people afterwards”*.

Stakeholder, Female, 40–49

More broadly, criminal behaviour of family members may burden households when grandparents or relatives are required to care for children while their parents are incarcerated. Instances of domestic violence may result in isolation, financial strain or a fear of going outside inhibiting access to food.

“If you’re on a single pension then all of a sudden it puts your budget over or if your daughter or son goes to prison then you become the carer of their kids. Well, Centrelink doesn’t know that. Often carer assistance isn’t given or granted. They have five people in a family and they are living on a widow’s pension.”

Stakeholder, Female, 40–49

#### 3.1.4. Cultural Food

AR respondents indicated cultural food, especially halal products, were often inaccessible, expensive or required extensive travel to obtain. Where halal products could not be sourced or were viewed as too expensive, some individuals would go without food on religious grounds.

“In Australia we have to find certain products like halal meats and it’s difficult to find. We have to go 40 km to find the halal meats … It’s a big problem … We have to travel a lot to get it. The Muslim people are struggling in most areas of Perth, because most of the shops don’t sell halal meat.”

At-risk female, 30–39

A stakeholder also indicated that their clients have difficulty obtaining cultural food (including halal foods).

#### 3.1.5. Transport

Individuals without access to a car must walk or use public transport, and are limited by the amount of food they could carry in a single trip. The inability to buy in bulk and the need to undertake frequent shopping trips was seen by respondents as increasing the cost associated with healthy food and meal preparation. Alternatively, respondents relied on friends or family members for travel, but this often required payment for fuel and prior planning.

“I find it difficult to get to the shops when I don’t have a car. Because you can’t get everything you want in one shop. I don’t have license so I have to walk everywhere.”

At-risk female, 20–29

#### 3.1.6. Illness and Mobility

Medications and doctors’ visits represented unexpected costs and made budgeting challenging. Those with mental illness could have intense anxiety or fear about going to shops/supermarkets that are heavily populated, and this could hinder a person’s ability to get nutritious food.

“I’ve got illness. Well I suffer from panic attacks, so I don’t like to go to the shops. I like to be able to see the exit and go quick and get out. I don’t want to go to the big shops.”

At-risk female, 50+

Illness and older age can be related to taste changes, decreased appetite and fatigue, resulting in reduced motivation to purchase and prepare food. Stakeholders expressed concern about the elderly and those with disabilities for who reduced mobility could impact their ability to obtain food.

### 3.2. Food Availability Pillar

Availability was the second most noted theme overall (18%, *n* = 59); 21% for the AR group (*n* = 43) and 13% for the stakeholder group (*n* = 16). Four sub-themes of food availability that emerged included price, location, quality, variety and water supply. These are discussed below. 

#### 3.2.1. Price

The majority (56%) of AR respondents considered food to be expensive or unaffordable. Fruit, vegetables and seafood were the most frequently cited as the least affordable foods; rice, flour, bread and “junk” foods were perceived to be the cheapest options. Understandings of healthy food varied within the AR group. Some individuals felt food needed to be organic (frequently a more costly option) to be considered healthy. Healthy foods, such as avocados and mangoes, were considered luxury items for the AR group due to their prohibitive price.

“When I first got here, I took a picture and put it on Facebook, because an avocado cost $3...”

At-risk female, 40–49

However, stakeholders primarily mentioned the “subsidization” of processed foods and referenced supermarkets using marketing strategies, such as “loss leaders” as incentives. Promotional tactics were cited as taking advantage of vulnerable populations and encouraging an obesogenic environment where overconsumption of high-energy food was the social norm. 

#### 3.2.2. Location

Both stakeholders and the AR group agreed location affected variation in the price, quality and variety of food. An inconsistency in the price of food was noted across Australian states, but respondents largely spoke about the disparity between metropolitan, regional and remote areas within Western Australia. Respondents who worked or had lived in regional or remote areas spoke about the struggle to obtain healthy food due to climatic conditions, distance and community store processes.

“… a local regional and remote community store … wasn’t providing any fresh fruit and vegetables because they thought it would be wasted, and there is a higher profit margin of white bread, sugar, coke and so on.”

Stakeholder, Female, 20–29

Stakeholders commented that food outlets were strategically located to take advantage of vulnerable groups.

“It’s the ease of fast food being available and they do it to prey on the weak (fast food company) put a (store) right at the corner of the park… They absolutely knew there were some very vulnerable people and very low economic consumers that could buy their $1 or $2 or whatever it costs burger and they put themselves absolutely purposely in that spot…We know that it’s mostly plastic cheese and disgraceful meat, but people eat it because it’s cheap...”

Stakeholder, Male, 40–49

AR respondents acknowledged that junk or fast food often provided a convenient and cost effective option. Opening hours and the distance to supermarkets were barriers to availability mentioned by the AR group. Shops and restaurants that closed by 9 p.m. limited the choice of those working late hours to expensive convenience stores or cheaper fast food options.

“… especially when I was living far away from a shopping centre, I mean there were times when I was so hungry that I would just pop down to the servo to grab a pie or something.”

At-risk male, 20–29

#### 3.2.3. Quality and Variety (Including Water Supply)

Both stakeholders and the AR group considered the variety and quality of food within the metropolitan area was generally good. Stock was perceived as superior in quality and variety when compared to food sold in regional areas. Imported and exported foods were front of mind for both those AR and stakeholders. The AR respondents felt imported foods were inferior and at times not safe to consume. 

Stakeholders were concerned about the net result of the export of healthy unprocessed foods and the subsequent import of processed non-nutritious foods.

“Because technically we have an abundant food supply, so it does come down to the individual, community and food policy side of things. There is such a great availability of non-nutritious foods.”

Stakeholder, Female, 30–39

The concept that food security was inclusive of the availability of both adequate safe food and drinks.

“It encompasses the adequate availability of water. We don’t have too much of an issue in metropolitan WA, but on the flip side how available the non-nutritious drinks are. Because that’s an area of concern as well’.”

Stakeholder, Female, 30–39

### 3.3. Food Utilisation Pillar

Overall, food utilisation was considered the third most significant driver of food insecurity (13%, *n* = 44); however, this pillar was perceived as more important for the stakeholder group (26%, *n* = 31) than for the AR group (6%, *n* = 13). Another notable point of difference was the primary sub-theme for each group; food literacy was the most referenced sub-theme for stakeholders while cooking facilities was paramount for the AR group. 

#### 3.3.1. Food Literacy

Stakeholders identified limited cooking skills or nutrition education as a food security barrier and current initiatives were often already preaching to the converted. Aggressive food marketing and advertising was viewed as affecting people’s food choices, especially those with either lower education levels or poor food literacy. There was also a perception that their clients could not afford to experiment or try new recipes, in fear of their families not eating the food and increasing food waste.

“I think the low food literacy is a big part of it. Teaching people about food budgeting and that fresh healthy food per kg is cheaper and then teaching them the skills to cook it. Also having enough food to meet their nutrient requirements. So if they are just getting the cheap take away or the sausages, white bread. They might have enough food, do they have enough nutrients.”

Stakeholder, Female, 30–39

The AR group largely did not view their food literacy skills as impacting their ability to obtain enough healthy food. Only the six male student respondents commented that their lack of confidence in cooking and nutrition knowledge had been an issue in the past. 

#### 3.3.2. Cooking Facilities and Resources 

Sharing cooking and storage facilities was highlighted by the AR group as a barrier to food security. For those at risk of food insecurity, a lack of, or unclean cooking facilities, made food preparation challenging and time consuming.

“I live in a communal place or area in a refuge. Everyone has to share the kitchen, so that makes it really hard. As opposed to having your own kitchen. I find it really frustrating, to be honest. I can’t wait to be out of there. Especially when you have a little kid and when other people aren’t clean. They just leave their stuff there and you have to work around it. It’s not your job to clean up after them, but they don’t do it as quickly as you do. I ended up saying something to someone yesterday and it ended up being a huge confrontation. It doesn’t really make you want to cook.”

At-risk female, 30–39

Stakeholders spoke about a general lack of very basic cooking facilities and how this ultimately impacted consumption of healthy food by people experiencing food insecurity.

“This affects Aboriginal people as well. Something like 40% of them doesn’t have a kitchen. Or a stable address either. And those who have home the equipment still doesn’t work. We see that a lot with our clients, a lot of them don’t have fridge so they can’t buy in bulk so they buy little bits and pieces. They basically have to use it all up within the day. Especially in the summer.”

Stakeholder, Female, 20–29

Additionally, stakeholders and the AR group agreed that cooking could be a large financial investment. For example, a lack of pantry staples makes cooking from scratch expensive, as all ingredients need to be purchased. People who move frequently may not be able to take pantry ingredients with them.

*The other thing is cooking a meal, when you have is a small amount of money. It’s relatively large investment to go to get everything you need, because you won’t use all of it in the meal and some of that doesn’t last all that long. So unless you’re cooking meals regularly it looks a lot worse on paper than the fast food options out there*.

At-risk male, 20–29

#### 3.3.3. Time 

Time as barrier to food security was only mentioned by the AR group. Careful budgeting, strategic shopping and healthy meal preparation were all considered to be time consuming tasks, which presented a challenge when individuals were already overcommitted.

“It requires you to make a lot of trips and you’ve got to be checking it out really often and there are people that won’t go to spend time for that.”

At-risk male, 20–29

### 3.4. Stability Pillar 

Stability (5%, *n* = 15) had the least number of themes emerged for stakeholders (12%, *n* = 14) and the AR group (<1%, *n* = 1). Policy and economic fluctuations were the sub-themes discussed.

#### 3.4.1. Policy

Stakeholders conceded that the current public perception was that obtaining healthy food was a personal choice. Stakeholders expressed concern that governments shifted excessive responsibility for obtaining healthy food onto individuals, and that too little attention was given to the role played by policy and industry.

“It’s like they have a choice, but they don’t actually have a choice. They don’t have access. It’s unavailable. It’s that sort of rhetoric of it’s their choice to eat healthier or not to budget. It’s kind of short changing all these other factors. It’s really a right wing platitude. They are just the ignorant mass.”

Stakeholder, Female, 50+

#### 3.4.2. Economic Fluctuations 

Stakeholders discussed the impact of the recent mining boom and bust in Western Australia on the gap between rich and poor. This gap increased as the prices went up with demand, but stakeholders also noted that when the mining boom stopped, the gap still existed as jobs were lost. The cost of living has continued to rise despite the reduction in wages and job losses.

*“Look, there are many reasons, with the economic downturn there are redundancies. There could be people that are very highly geared, so that they were thinking that the mining boom or the gravy train was just going to continue. They might have just got the big house and lots of cars. You know completely geared themselves to have this lifestyle and possibly it fell unstuck that way”*.

Stakeholder, Female, 30–39

When prompted, all of the AR respondents agreed that the cost of living had remained high despite the resulting economic downturn.

## 4. Discussion

There was general agreement between people at risk of food insecurity and stakeholders working in the area on many of the factors considered to be driving food insecurity in Western Australia. Of interest was the most apparent deviation in regard to the relative importance attributed to each of the pillars of food security. The stakeholders tended to focus on big-picture concepts outlined in the Stability and Availability pillars, including government policy and changes to the economic climate, whilst those at risk spoke of their personal experience and the minutiae impacting their daily lives, such as prioritisation of income, found within the Access pillar. These diverse themes emerging from two different perspectives, the stakeholders and the AR group, are consistent with the findings reported by other Australian studies [11,23] and similar to trends found between stakeholders and food-insecure individuals in Canada [8]. 

Study respondents unanimously considered adequate income as an essential component for food security and that current government welfare allowances were insufficient. Indeed, poor resourcing has been documented by several studies as the principal barrier to healthy food acquisition [23,24,25]. However, a point of difference between the respondent groups shown here was the perception of the prioritisation of healthy food and the acknowledgement of price as an obstacle. In concordance with the findings of Hamelin, Mercier and Bédard [8] AR respondents noted both the importance of and desire to eat a healthy diet, but simultaneously perceived healthy foods to be unaffordable and beyond their reach. This concept was previously reported by Laurel, et al. [26], who found that Australians from the lowest income bracket were required to spend an estimated 50% of their income to eat in accordance with the recommended dietary guidelines. This amount is above a recently calculated marker of food stress, which occurs when 25% or more of income is spent on food [27]. Previous research has suggested that when the cost of fruit and vegetables is subsidised, the consumption of these foods increases within low-income households [28]. This implies that the perceived prohibitive cost of healthy food is a significant challenge, regardless of a person’s desire or motivation. Additionally, AR respondents and other research have cited non-financial factors such as distance to shops [29] and lack of car ownership [3] that could quite literally put healthy food beyond the reach of some individuals. 

A characteristic feature of food insecurity without hunger in developed nations is the dietary compromise of quantity over quality that typically results in the overconsumption of high-energy foods [30,31]. Ultimately, the AR respondents followed this trend, as they prioritised a feeling of fullness over the nutritional quality of food, resulting in a less nutritious diet. The stakeholders in this study, however, chiefly spoke about the overabundance of cheap, energy-dense products displacing the intake of nutritious foods in food-insecure people and the expense of healthy food was less frequently cited.

Another important disparity between the respondent groups was the identification of the main challenges, largely within the Utilisation pillar, to meal preparation. Barriers to cooking healthy meals reported in the literature and in this study include the expense of pantry items [32], the inability to obtain cultural foods [33] and poor access to kitchen or food storage facilities [11]. The AR respondents cited kitchen facilities as the greatest obstacle to cooking, while stakeholders focused more on food literacy (cooking and nutrition) skills. 

“Food literacy is the ability of an individual to understand food in a way that they develop a positive relationship with it, including food skills and practices across the lifespan in order to navigate, engage, and participate within a complex food system” [34]. Food literacy is encompassed within the utilisation pillar and was regarded by the stakeholders as an important means to assist individuals ameliorate some the effects of food insecurity and improve their ability to navigate the complex food system [35,36]. This is in contrast with the current North American position, where nutrition interventions targeting food-insecure people are thought to be ineffective, as their limited income does not allow for the implementation of healthy eating principles [8,37,38]. In our study, only the male AR respondents believed their food literacy skills had impacted their intake of healthy food, whereas, female AR respondents felt they were very resourceful and used a large range of coping strategies to reduce the impact of a limited income on food intake. Furthermore, McLaughlin, et al. [39] found no association between food security status in Canadian women and the frequency of cooking from scratch. Food-insecure women, however, tended to prepare less complex recipes, possibly due to the expense of the ingredients rather than a lack of skills. However, the researchers in this study observed varied levels of nutrition knowledge among their AR group. For example, many AR respondents believed food needed to be organic to be considered healthy. It is therefore plausible that people with less severe food insecurity could benefit from an improved understanding of what constitutes a healthy diet. Regardless, stakeholders within this study also noted differences associated with gender; in particular food preparation, shopping and cooking primarily seemed to follow traditional gender roles and was largely the responsibility of women [31,40]. Studies have demonstrated that men living alone may lack basic cooking and nutrition skills, preventing them from adequately addressing their own eating requirements [40,41]. It is therefore possible that limited food literacy skills could be a barrier to food security for males. However, due to low representation of males in our study generalising these results should be cautioned, and more research is warranted focusing on the male perspective.

Emerging themes in our study also included the impact of poor health and the effect of the prioritisation of alcohol and drugs on food security status. Both respondent groups agreed that these issues were detrimental to a person’s food security and competed with cost of medical appointments, medications and healthy food. Previous research shows positive associations between poor health, particularly mental health [17], and tobacco [42] and drug usage [43,44] with food insecurity. To date it is unclear whether these associations are causal or a symptom of low socioeconomic status. 

The strength of this study was the consideration of viewpoints from both stakeholders and a variety of people at risk of food insecurity. Focus groups were conducted to code or data saturation and therefore provided a well-rounded depiction of the issues. A limitation of this study was an overrepresentation of female and tertiary student respondents and an underrepresentation of several subgroups (regional and remote, Aboriginal people). Therefore, our study population was not a representative sample of the Australian population and this limits the generalization of the results. Additionally, due to concerns about literacy skills, a food security measurement was not applied to the AR respondents. The researchers could therefore not definitively classify these individuals as “food-insecure” at the time of focus group or interview. However all participants in the AR focus group gave examples of food insecurity, which provided adequate justification for their classification. 

### Recommendations to Assist Stakeholders to Better Combat Household Food Insecurity

Based on the findings of this research the following recommendations have been suggested:

1. Include pantry items and culturally appropriate foods in emergency food relief packs.
2. Assist individuals to cook meals at home by subsidizing the cost of healthy foods, particularly fruit and vegetables.
3. Tailor food literacy programs to more effectively engage males.
4. Provide education for stakeholders that motivation or desire may not be the underlying reasons for overconsumption of non-nutritious foods in food-insecure people, and that other factors such as prioritization of funds or time may be an issue.

## 5. Conclusions

Although there was agreement on the perceived importance of the major drivers of food insecurity, such as income, there were notable differences between the AR individuals and stakeholders when it came to some factors, including time, food literacy, health policy and the price of food. These differences may in part be attributed to the varied role each group has to play in the resolution of the problem. It is understandable that stakeholders would focus their efforts on lobbying for enhanced government and industry policy as this would enact significant change at a system level. However, it is still important to acknowledge the lived experience of food-insecure individuals and recognize that a lack of motivation or desire may not be the underlying reason for poor dietary intake. 

## Figures and Tables

**Table 1:** Characteristics of stakeholders (*n* = 13).

Characteristic	*n*	%
Age (years)		
20–29	4	31
30–39	2	15
40–49	3	23
50+	4	31
Sex		
Female	11	85
Education		
<Tertiary	1	8
Tertiary	12	92
Employer		
Not for profit	9	69
Government	1	8
Academic institution	3	23

Tertiary = obtained a tertiary qualification (e.g., diploma, academic degree).

**Table 2:** Characteristics of ‘at risk’ (*n* = 34).

Characteristic	*n*	%
Age (years)		
20–29	15	44
30–39	6	17
40–49	5	15
50+	8	24
Gender		
Female	28	82
Education		
<Tertiary	25	74
Tertiary	9	26
Aboriginal and Torres Strait Islander	3	9
Culturally and Linguistically Diverse	10	29

Tertiary obtained a tertiary qualification (e.g., diploma, academic degree).

## Supplementary Materials

The following are available online at http://www.mdpi.com/2072-6643/10/8/1059/s1, File S1: Interview guide and research questions.
