# Supplementary material for: What Drives Food Insecurity in Western Australia? How the Perceptions of People at Risk Differ to Those of Stakeholders
Source: Nutrients. 2018 Aug 9;10(8):1059. doi: 10.3390/nu10081059 (PMC6115936; doi:10.3390/nu10081059)
Supplement: Supplementary file 1 [file nutrients-10-01059-s001.pdf]

Interview guide*

Wording and language may have varied depending on the type of group and literacy level of the participants.

**Focus group objectives**<br>1. To determine the experiences and coping strategies of food insecure individuals in Western Australia.<br>2. To examine the beliefs, values and attitudes of food insecure individuals and stakeholders in relation to food acquisition.<br>3. To investigate perceived barriers/challenges and enablers for food insecurity in Western Australia.

Food security is when you have enough good food for your needs.

Food insecurity is when you don't have enough of the types or amounts of foods you need. This problem could be due to lack of money, the shops don't sell healthy foods or transport to purchase food.

	Obj 1	Obj 2	Obj 3
**Is food insecurity OR access and availability to food a problem in your community?**	**x**		
How big is the problem for you?	x		
How big is the problem for others around you?	x		
**Is food in Western Australian affordable and available?**	**x**	**x**	**x**
- How is it or is it not?		x	x
- Are there differences between Perth and country/ regional areas?		x	x
**What are barriers or challenges to getting enough food?**			**x**
- Why do you think these exist?			x
- How does transport impact your feeling of getting access to food?			x
Is it about not getting enough food OR about getting good quality food?<br><br>Would you like that to be different?<br><br>What could make it different for you?	x	x	
**Have you had experience any experience with food insecurity??**	**X**		
Do you know anyone who struggles to get enough good food?	x		
**What are your priorities when spending money?**<br>**Can you order these?**		**x**	
Is healthy food a priority?<br>Or just any food a priority?		x	

1. Using butchers paper or stick notes or pictures, rate the top three priorities to spend money on?

For example- Food, Bills, Medications

Introduction

1. Thank you for coming tonight/ today and giving up your time.
2. My name is XX and I am a researcher with XX University.
3. Today/ tonight we would like to gain your thoughts and perspectives about food security. There are no right or wrong answers; we would simply like to gain your views. If you feel differently to others, please speak up, we/ I would like a full range of views so don't be afraid to say something different to everyone else. All results from this focus group will be anonymous.
4. We/ I would like to hear from everyone and make sure everybody gets a chance to have their say, so please be respectful of other people's opinions and 'let everyone have a fair go'.
5. I/We would like you to feel comfortable about being open and ask that you treat anything that is said within the group as confidential.
6. Unless you may be needed urgently, please can you turn your mobile phone off or to silent?
7. And also, just to mention that we are audio-taping the session for research purposes.
8. I'd like to begin by defining food security.

Other tasks to be completed to prior to commencement

- Warm up, participants to introduce themselves
- Informed consent
- Housekeeping – bathrooms, timing, emergency exits, refreshments

Conclusion

1. Think we might begin to wrap up now. Can I ask first, does anyone have anything burning that they would like to say? Or something that they thought of, but there wasn't the opportunity to say it or the opportunity passed?
2. Thank you to you all for you time and sharing. We really appreciate it. Information gained from tonight will be used to improve our knowledge about food insecurity. If you would like some further information on the project, we have put together a small pack that you can take away with you if you wish. Results of the project can also be available to you, just let me know if you are interested. Also, if you have any friends or family who might like to be involved in this research, please get them to give me a call. Though I do ask that if you have any friends who are coming to one of our focus groups that you do not disclose the topic of interest as we are keen to see how this topic naturally comes up within the discussion.
3. Just lastly, I would like to again emphasise the importance of treating everything that was discussed as confidential.
